# Proteogenomics identifies c-Met inhibition as a therapeutic strategy for BAP1-deficient clear cell renal cell carcinoma

**DOI:** 10.1186/s43556-024-00220-z

**Published:** 2024-11-12

**Authors:** Bowen Du, Yulin Zhou, Wenzhi Li, Haowei He, Ming Chen, Ninghan Feng

**Affiliations:** 1 Department of Urology, Jinling Hospital, Affiliated Hospital of Medical School, Nanjing University, Nanjing, China; 2 Department of Urology, Shanghai General Hospital, Shanghai Jiao Tong University School of Medicine, Shanghai, China; 3 Department of Urology, Affiliated Zhongda Hospital of Southeast University, Nanjing, China; 4 Department of Urology, Jiangnan University Medical Center, Wuxi, China

Dear editor

Kidney cancer ranks among the top ten most frequently diagnosed cancers. Clear cell renal cell carcinoma (ccRCC) is the predominant subtype of kidney cancer. The genetic heterogeneity of ccRCC tumors frequently leads to resistance to tyrosine kinase inhibitors (TKIs) and immunotherapies. Personalized therapies based on molecular subtypes are emerging as a strategy for ccRCC treatment. BRCA1-Associated Protein 1 (BAP1) mutation, associated with TKI resistance [1], defines a subgroup of ccRCC, encompassing approximately 10% of ccRCC patients. However, therapeutic options for BAP1-mutated ccRCC have not been well studied. We enrolled 72 ccRCC patients and conducted comprehensive genomic, transcriptomic, and proteomic profiles on tumor specimens alongside paired normal adjacent tissues (NATs) from these patients. The principal component analysis identified abnormal proteomic data of one ccRCC tumor (outlier). Consequently, the following analysis proceeded with data from the remaining 71 patients.

Previous studies have highlighted the pivotal role of the tumor microenvironment in ccRCC treatments. To gain insight into features of immune infiltration in ccRCC, we analyzed the tumor microenvironment across all samples with deconvoluted cell signatures using xCell. We classified these samples into six major subtypes and one minor subtype containing only two tumors. As shown in Fig. 1a, the six major subtypes are (1) Hot-Treg, characterized by enrichment of Treg cells and Th2 cells; (2) Hot-M1, characterized by enrichment of M1-like tumor-associated macrophages (TAMs) and CD8^+^ T cells; (3) Hot-M2, characterized by enrichment of M2-like TAMs; (4) Cold-Epi, characterized by enrichment of epithelial cells; (5) Cold-Endo, characterized by enrichment of endothelial cells; and (6) Cold-Mix, characterized by enrichment of endothelial cells and myocytes. The Hot-M2 subtype exhibits the highest proportion of BAP1 mutations (*p* = 0.013), indicating a potential link between BAP1 mutations and M2-like TAMs. Additionally, BAP1-mutated tumors present higher TAM scores (fold change = 4.68, *p* < 0.001) and M2-like TAM scores (fold change = 4.08, *p* = 0.018). These findings suggest that BAP1 mutation elicited the alteration of immune microenvironment in ccRCC.

**Fig. 1:**
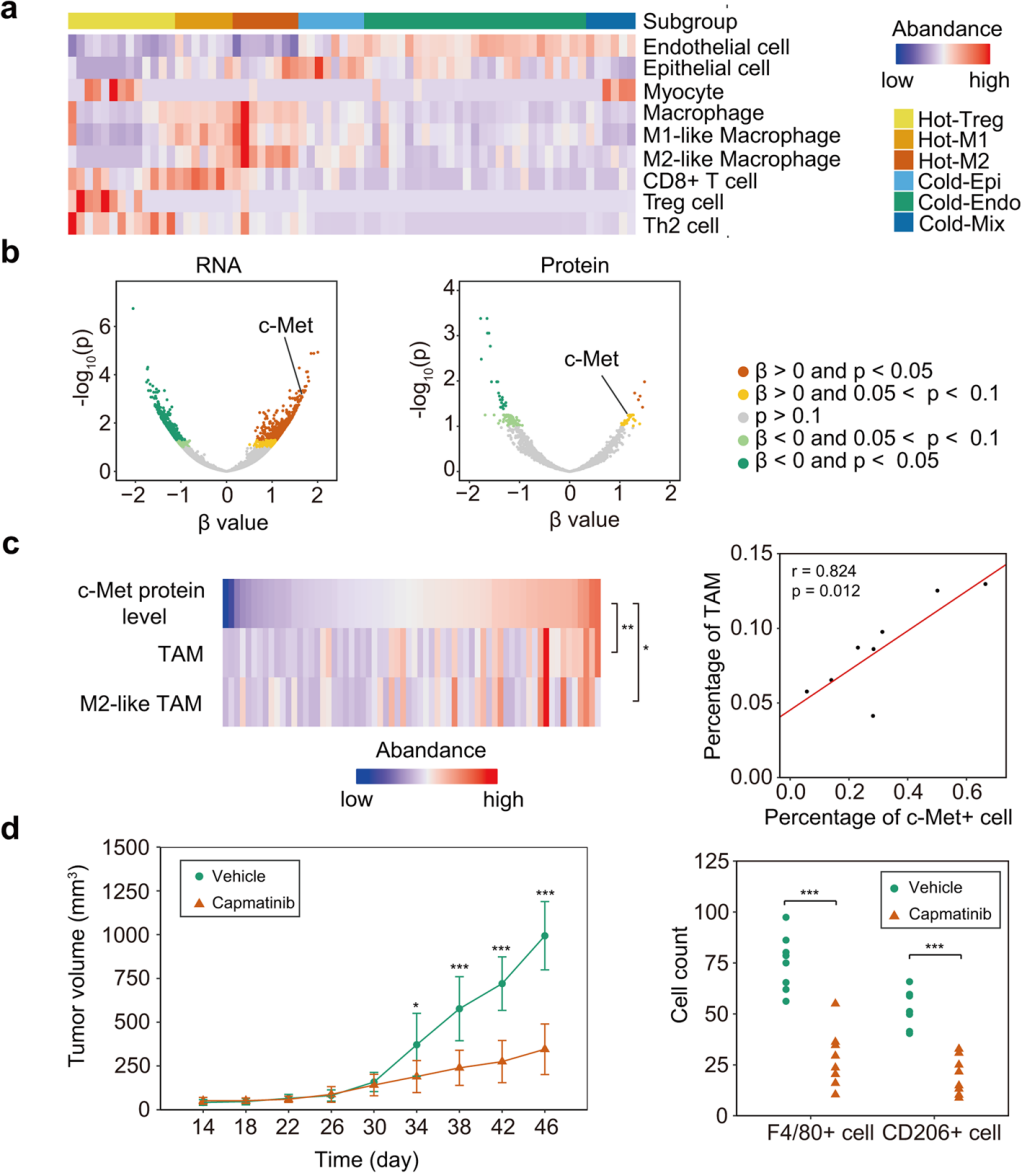
Proteogenomic analysis reveals c-Met as a therapeutic target in BAP1-mutated ccRCC. **a** Immune subtypes of ccRCC tumors. The heatmap represents the relative abundance of nine types of cell signatures in the six major immune subtypes. The six major immune subtypes include Hot-Treg, Hot-M1, Hot-M2, Cold-Epi, Cold-Endo and Cold-Mix. **b** Gene expression associated with BAP1 mutation. Gene expression associated with BAP1 mutation was analyzed with the linear regression model adjusted to tumor purity. The coefficients (β values) represent the association between RNA or protein levels and BAP1 mutation. Positive β values indicate positive association between gene expression and BAP1 mutation. Negative β values indicate negative association between gene expression and BAP1 mutation. c-Met expression is positively associated with BAP1 mutation. **c** Left: association between c-Met expression with TAMs and M2-like TAMs. The heatmap represents the relative abundance of c-Met, TAMs, and M2-like TAMs. c-Met level is positively correlated with abundance of TAMs and M2-like TAMs. p-value was calculated with the Spearman correlation. *p < 0.05, **p < 0.01. Right: association between the percentage of c-Met + ccRCC cells and the percentage of TAMs. p-value was calculated with the Spearman correlation. **d** Left: volume of BAP1-KO RENCA syngeneic tumors. BAP1-KO RENCA cells were subcutaneously injected into BALB/c mice and treated with the c-Met inhibitor (Capmatinib) or vehicle (*n* = 8 per group) at day 14. Tumor volume was measured every four days. The Student’s t test was used to measure the difference of volume at every time point. Capmatinib inhibits the growth of syngeneic tumors. *p < 0.05, ***p < 0.001. Right: number of F4/80 + cells and CD206 + cells in BAP1-KO RENCA tumors treated with Capmatinib or vehicle. Infiltration of F4/80 + TAMs and CD206 + M2-like TAMs was inhibited by Capmatinib. p-value was evaluated with the Wilcoxon test. ***p < 0.001

To elucidate the impact of the BAP1 mutation on ccRCC development and microenvironment remodeling, we conducted a comparative analysis of gene expression profiles between BAP1-mutated and BAP1-WT tumors, utilizing regression analysis that was adjusted to tumor purity (Fig. 1b). Our results highlight a significant association between c-Met expression and BAP1 mutation at both RNA (β = 1.65, FDR = $\:7.9\:\times\:{10}^{-4}$) and protein levels (β = 1.17, FDR = 0.057, Fig. 1b). Moreover, Chen et al. explored proteomic alterations induced by BAP1 knockout in a ccRCC cell line [2]. Re-evaluation of these data revealed that BAP1 knockout leads to increased c-Met protein levels (*p* < 0.001). These findings propose that c-Met might be a pivotal mediator in ccRCC progression and microenvironment remodeling driven by BAP1 mutation.

Tumor microenvironment and immune molecular influence the outcome of immune therapy [3]. In this study, we investigated the role of c-Met in ccRCC microenvironment remodeling. Previous studies have highlighted the accumulation of TAMs in ccRCC tumors resistant to immune checkpoint inhibitors (ICIs) [4], positing a potential impact of TAMs on the response to ICIs. However, the mechanisms underlying TAM infiltration in ccRCC tumors remain largely unexplored. Our study reveals a significant correlation between the c-Met protein level and the abundance of TAMs (*p* = 0.0017) and M2-like TAMs (*p* = 0.014) in our ccRCC cohort (Fig. 1c, left panel). Similarly, a significant correlation between the c-Met protein level and TAM abundance was validated in the Clinical Proteomic Tumor Analysis Consortium (CPTAC) cohort (β = 8.3790, *p* = 0.0041). Re-analysis of existing single-cell RNA sequencing (sc-RNA-seq) data [5] supports the correlation between c-Met^+^ ccRCC cells and the abundance of TAMs (Fig. 1c right panel). Moreover, our study demonstrated that the c-Met protein level correlates with the RNA levels of CSF1 (*r* = 0.339, *p* = 0.005), IL-10 (*r* = 0.462, *p* < 0.001), and CCL18 (*r* = 0.347, *p* = 0.004), which are critical in macrophage recruitment and polarization [6]. These findings indicate that c-Met may be responsible for TAM infiltration by stimulating tumor cells to express the associated cytokines and chemokines.

We further evaluated the influence of c-Met on immune therapy outcomes in ccRCC. Re-evaluating sc-RNA-seq data from ICI-treated ccRCC tumors [4] revealed that ICI-resistant tumors exhibited a higher proportion of c-Met^+^ cells and TAMs but a lower ratio of CD8^+^ T cells (Fisher’s exact test, *p* < 0.001). Moreover, we re-analyzed previous clinical trials (CheckMate-9, -10, and − 025) [7] and found that Nivolumab-treated BAP1-mutated patients with high c-Met levels exhibit worse overall survival (OS, log-rank test, *p* = 0.01) and progression-free survival (PFS, log-rank test, *p* < 0.001) than those with low c-Met levels. However, we detected no difference in OS (log-rank test, *p* = 0.2) or PFS (log-rank test, *p* = 0.9) between the c-Met-high and -low Nivolumab-treated BAP1-WT patients. We also noticed that TAM abundance inversely correlates with CD8^+^ T cell abundance in highly immune cell infiltrated tumors in our cohort (*r* = -0.725, *p* = 0.027); however, this correlation did not extend to all tumor samples. This disparity may be due to TAMs influencing T-cell infiltration only above a concentration threshold. Given the observed c-Met-driven TAM infiltration, we postulate that c-Met may attenuate ICI therapy efficacy through TAM-induced CD8^+^ T cell suppression.

Scientists have developed multiple research tools for urological malignancy [8, 9]. To substantiate our findings, we employed a syngeneic mouse model, subcutaneously injecting either BAP1-knockout (BAP1-KO) or control (BAP1-Ctrl) mouse renal adenocarcinoma cells (RENCA) into BALB/c mice. We validated BAP1 knockout with Sanger DNA sequencing in cell lines and immunohistochemical (IHC) staining in syngeneic tumors. We administered Capmatinib, a highly specific c-Met inhibitor, to the tumor-bearing mice. We noted that BAP1-KO tumors under c-Met inhibition showed significantly lower volumes than untreated counterparts (Fig. 1d left panel). Conversely, this inhibitory effect was not observed in BAP1-Ctrl tumors, as no significant difference in tumor volume was detected throughout the 50-day Capmatinib treatment. These findings suggest that c-Met inhibitors may suppress the growth of BAP1-mutated ccRCC tumors.

We further assessed TAM and M2-like TAM infiltration in the syngeneic tumors using IHC staining for F4/80^+^ TAMs and CD206^+^ M2-like TAMs. We observed a lower abundance of F4/80^+^ TAMs and CD206^+^ M2-like TAMs in BAP1-KO tumors treated with Capmatinib than their untreated BAP1-KO counterparts (Fig. 1d right panel). However, CD8^+^ T cell infiltration, assessed through IHC staining for CD3 and CD8, was not observed in both BAP1-KO and BAP1-Ctrl tumors, which may be attributed to the weak T cell response elicited by subcutaneous RENCA tumors. Our syngeneic mouse model supports our primary finding that c-Met may be critical in promoting cancer progression and TAM infiltration in BAP1-mutated ccRCC.

This study delineates the molecular features of ccRCC tumors and explores the therapeutic strategy for BAP1-mutated ccRCC through multi-omics analysis and syngeneic mouse models. We established c-Met as a mediator between BAP1 mutation and ccRCC tumor growth and microenvironment remodeling. The mouse model revealed that c-Met inhibition hinders ccRCC tumor growth and TAM infiltration in BAP1-mutated ccRCC. Our study reveals the mechanism of cancer progression and microenvironment remodeling in BAP1-mutated ccRCC, highlighting its significant clinical implications by identifying a potential therapeutic target for improving treatment strategies.

## Supplementary Information

Supplementary Material 1.

## Data Availability

The data of this study are available as reasonable consultation with the corresponding authors.

## Abbreviations

AKT1: AKT Serine/Threonine Kinase 1
BAP1: BRCA1 Associated Protein 1
CCL18: C-C Motif Chemokine Ligand 18
CSF1: Colony Stimulating Factor 1
JAK1: Janus Kinase 1
KDM5C: Lysine Demethylase 5C
PBRM1: Polybromo 1
PLCγ: Phospholipase C Gamma 1
PTEN: Phosphatase and Tensin Homolog
PTK2: Protein Tyrosine Kinase 2
SETD2: SET Domain Containing 2
VHL: Von Hippel-Lindau Tumor Suppressor
