# Supplementary material for: Proteogenomics identifies c-Met inhibition as a therapeutic strategy for BAP1-deficient clear cell renal cell carcinoma
Source: Mol Biomed. 2024 Nov 12;5:56. doi: 10.1186/s43556-024-00220-z (PMC11555054; doi:10.1186/s43556-024-00220-z)
Supplement: Supplementary file 1 — Supplementary Material 1. [file 43556_2024_220_MOESM1_ESM.docx]

Proteogenomics Identifies c-Met Inhibition as a Therapeutic Strategy for BAP1-Deficient Clear Cell Renal Cell Carcinoma

Bowen Du^1, †, *^, Yulin Zhou^1, †^, Wenzhi Li^2, †^, Haowei He^1, *^, Ming Chen^3, *^, Ninghan Feng^4, *^

^1^Department of Urology, Jinling Hospital, Affiliated Hospital of Medical School, Nanjing University, Nanjing, China

^2^Department of Urology, Shanghai General Hospital, Shanghai Jiao Tong University School of Medicine, Shanghai, China

^3^Department of Urology, Affiliated Zhongda Hospital of Southeast University, Nanjing, China

^4^Department of Urology, Jiangnan University Medical Center, Wuxi, China

^†^These authors contributed equally: Bowen Du, Yulin Zhou, Wenzhi Li

^*^Correspondence:

Bowen Du

[dubwac@gmail.com](mailto:dubwac@gmail.com)

Haowei He

[hohowell@163.com](mailto:hohowell@163.com)

Ming Chen

[mingchen0712@seu.edu.cn](mailto:mingchen0712@seu.edu.cn)

Ninghan Feng

[n.feng@njmu.edu.cn](mailto:n.feng@njmu.edu.cn)

**Materials and methods**

**Sample collection and clinical annotation**

The ccRCC tumor tissue samples and paired normal adjacent tissues (NATs) were obtained from Jinling Hospital. These samples were chosen regardless of their clinical stages or histologic grades. All samples were re-coded, replacing the name and hospital number of the patients. This study was approved by the Research Ethics Committee of Jinling Hospital, and written informed consent was obtained from each patient. Clinical data were sourced from Jinling Hospital. Cases were graded using the Fuhrman Nuclear Grading System and staged based on the AJCC cancer staging system (7th edition). To assess tumor cellularity, formalin-fixed, paraffin-embedded sections corresponding to the ccRCC tissue samples were examined. Hematoxylin and eosin (H&E)-stained slides were independently reviewed by at least two experienced pathologists. Tumor cellularity refers to the proportion of tumor cells in a tissue sample. Tumors with cellularity below 80% were excluded.

**WES data analysis**

DNA was extracted from tissues using the DNeasy Blood & Tissue Kit following the manufacturer’s instructions. WES and library construction was performed using the Agilent SureSelect Human All Exon V6 Kit. After cluster generation, the DNA libraries were sequenced on the Illumina NovaSeq 6000 platform. WES sequencing reads were aligned to the GRCh38 reference genome available on the TCGA website using the Burrows-Wheeler Aligner (BWA). The aligned reads were sorted with samtools, and BAM files from identical samples were merged using sambamba. PCR duplicates were eliminated with sambamba. For stringent quality control, reads were filtered with samtools and sambamba. Only de-duplicated, primary alignment, and proper pair reads advanced to subsequent analysis stages. Realignment of all insertions and deletions (Indels) was conducted using the RealignerTargetCreator tool from GATK, and base quality was recalibrated with the BaseRecalibrator tool from GATK. Somatic variants (SNV and Indel) were identified using Mutect2 from GATK. Briefly, variants in NATs were detected using Mutect2 in tumor-only mode, and the panel of normal (PON) was established using the CreateSomaticPanelOfNormals tool from GATK based on variants in NATs. Subsequently, somatic variants were identified using Mutect2 with exome data of tumor samples and their matched NATs. Strictly, variants were retained if they met these conditions: (1) mutations passed the Mutect2 filter; (2) at least five reads harboring the mutations; (3) the mutation allele frequency was more than 5%. Variants were annotated by ANNOVAR based on the RefSeq gene model.

**RNA-seq data analysis**

RNA was isolated using the Trizol reagent according to the manufacturer’s instructions. Sequencing libraries were generated using the NEBNext Ultra RNA Library Prep Kit for Illumina. After cluster generation, the cDNA libraries were sequenced on the Illumina NovaSeq 6000 platform. RNA-seq reads were aligned to the reference genome (GRCh38) and annotation files (Gencode v22) obtained from the TCGA website. The alignment process was executed using the STAR program in two-pass mode. Raw count data for each gene were calculated using HTSeq, and the FPKM value was derived by adjusting the raw count value to gene length and the total counts mapped across all genes.

**Proteomic data analysis**

Sample preparation for proteomic MS/MS analysis was performed according to a previous study [1]. The peptides were subjected to a capillary source and analyzed by timsTOF Pro mass spectrometry in PASEF mode. The electrospray voltage applied was 2.0 kV. Precursors and fragments were analyzed at the TOF detector, with an MS/MS scan range from 100 to 1700 m/z. Precursors with charge states 0 to 5 were selected for fragmentation, and 10 PASEF-MS/MS scans were acquired per cycle. The dynamic exclusion was set to 30 seconds. The MS/MS data were processed using MaxQuant (v.1.6.15.0), referencing the UniProt database comprising 20,380 sequences. A decoy database was used to calculate false discovery rates (FDR). The enzyme specificity was set to trypsin. The maximum missing cleavage site was set to 2. The fixed modification was set to Carbamidomethylation on cysteine. The variable modification was set to Acetylation on protein N-terminal and oxidation on methionine. The minimal peptide length was set to 7. The maximum number of modifications per peptide was set to 5. The mass tolerance for precursor ions was set to 20 ppm in the first search and 20 ppm in the main search. The mass tolerance for fragment ions was set to 20 ppm. The threshold of FDR was set to 0.01. For quantitative evaluation, the iBAQ value extracted from the MaxQuant results was used as the protein level for each gene. The batch effect was corrected using the ComBat function in the sva R package. To equalize protein input variations across samples, quantile normalization was applied using the normalize.quantiles function from the preprocessCore R package.

**Immune subtype identification**

The tumor purity and the immune scores for each sample were assessed using the ESTIMATE R package, based on RNA-seq data. The relative abundance of 64 different cell types was computed via the xCell R package. To identify the immune subtypes of ccRCC, consensus clustering was conducted on 64 cell types using the ConsensusClusterPlus R package. The clustering was performed using the “Pearson” method and the “Euclidean” distance. The total number of subsampling iterations was 1,000. The proportion of samples selected was 80% in each resampling. The range for the number of clusters considered spanned from 2 to 8. A consensus matrix with k = 7 displayed the most distinct separation between the clusters.

**Gene expression associated with BAP1 mutation**

The association between gene expression and BAP1 mutation was analyzed using the following regression model:

$mRNA \sim BAP1 mutation+covariates$,

$$protein \sim BAP1 mutation+covariates$$

Given that ccRCC is characterized by high immune and stromal infiltration, tumor purity was included as a covariate. A positive β value indicates overexpression in BAP1-mutated tumors, whereas a negative β value denotes underexpression in BAP1-mutated tumors. P-values were adjusted using the Benjamini-Hochberg method.

**c-Met expression in BAP1 knockout cell line**

Previous research investigated the effects of BAP1 knockout on gene expression in a ccRCC cell line (786O). Proteomic profiling was conducted on both BAP1-KO and control 786O cells. The proteomic data were sourced from the PRoteomics IDEntifications (PRIDE) database (https://www.ebi.ac.uk/pride/) with the dataset identifier PXD012288. The level of c-MET protein was assessed based on its abundance ratio. A Student’s t-test was employed to determine the difference between BAP1-KO and control 786O cells.

**Association of c-Met level with TAM and M2-like TAM**

The association of c-Met level with TAM and M2-like TAM was assessed using regression analysis. The models used were as follows:

$TAM\sim c-Met level+covariates$,

$$M2-like TAM\sim c-Met level+covariates$$

In these analyses, tumor purity served as the covariate.

**Single-cell RNA-seq (scRNA-seq) analysis**

In this study, two scRNA-seq datasets were employed. The first dataset, focused on ICB-treated ccRCC patients, which encompasses mix-response, complete-response, and resistant tumors, was downloaded from <https://trace.ncbi.nlm.nih.gov/Traces/index.html?view=analysis&acc=SRZ190804>. Cell type annotations were directly retrieved from the original file within the dataset. We calculated the rates of c-Met^+^ ccRCC in the total ccRCC population, TAM in the total cell population, and CD8^+^ T cell in the total cell population based on the cell type annotations. Fisher's exact test was utilized to evaluate the differences in rates of c-Met^+^ ccRCC, TAM, and CD8^+^ T cells across distinct tumors. The second dataset, encompassing data from 11 ccRCC patients, was obtained from <https://data.mendeley.com/datasets/nc9bc8dn4m/1>. Three patients lacking CD45- cells were excluded. For the remaining eight patients, cell types were reanalyzed using the Seurat (version 4) R package. Cell types were annotated according to the guidelines from the previous study. The rate of c-Met^+^ ccRCC in the total ccRCC population and the rate of TAM in the CD45^+^ cell population were computed. The correlation between the rate of c-Met^+^ ccRCC and the rate of TAMs was assessed using the Spearman correlation method.

**Survival analysis**

CheckMate-9, -10, and -025 data were reanalyzed by Braun et al. and obtained from the website (https://www.nature.com/articles/s41591-020-0839-y#Sec27). The Nivolumab-treated patients were selected and classified into BAP1-mutated and -WT groups. Each group was divided into the c-Met-low and -high groups according to the median of c-Met. The difference in survival between c-Met-low and -high groups was analyzed with a log-rank test using the survival R package.

**Mice and in vivo studies**

The RENCA cell line (ATCC, CRL-2947) was obtained from the Cell Bank of the Chinese Academy of Sciences. To ablate the mouse BAP1 gene in RENCA cells, specific guide RNA (gRNA) sequences were designed (Forward 5′- CAC CGT GTC AAA GGG GTG CAA GTG G -3′; Reverse: 5′- AAA CCC ACT TGC ACC CCT TTG ACA C -3′) targeting the mouse BAP1 gene. The Male BALB/c mice (5-6 weeks old) were obtained from the Ziyuan Experimental Animal Corporation and utilized for a subcutaneous syngeneic model according to prior protocol [2]. A suspension of 2 × 10^6^ viable cells was subcutaneously injected into the flank of mice. On the 14th day post-injection, the mice were orally administered 30 mg/kg of Capmatinib reconstituted in 0.5% methylcellulose and 0.05% Tween 80 once daily. Tumor volumes were tracked every four days using the formula $V=length \times{width}^{2}/2$. Mice with RENCA-BAP1-Crtl tumors were sacrificed at 50 days. Mice with RENCA-BAP1-KO were sacrificed at 46 days. Collected tumor samples were fixed in formalin overnight and embedded in paraffin. Immunohistochemical staining is performed according to the previous study [2] using the following antibodies: anti-BAP1 (ab255611, Abcam), anti-c-Met (AF527-SP, R&D), anti-F4/80 (AF527-SP, CST), and anti-CD206 (24595T, CST). All mouse experiments were approved by the institutional review board and ethics committee of Shanghai General Hospital.

**Supplementary reference:**

1. Li Y, Yang B, Ma Y, Peng X, Wang Z, Sheng B, et al. Phosphoproteomics reveals therapeutic targets of esophageal squamous cell carcinoma. Signal Transduct Target Ther. 2021;6(1):381.
2. Fu Q, Xu L, Wang Y, Jiang Q, Liu Z, Zhang J, et al. Tumor-associated Macrophage-derived Interleukin-23 Interlinks Kidney Cancer Glutamine Addiction with Immune Evasion. Eur Urol. 2019;75(5):752-763.
